# Children′s preoperative stress according to the parental presence evaluated by salivary cortisol and mYPAS: quasi-randomized trial^*^

**DOI:** 10.1590/1980-220X-REEUSP-2023-0232en

**Published:** 2024-03-08

**Authors:** Ketillyn Nayara de Macedo Silveira, Rodrigo Leal Alves, Paulo do Nascimento, Monique Antonia Coelho, Guilherme Antonio Moreira de Barros, Norma Sueli Pinheiro Módolo

**Affiliations:** 1 Universidade Estadual Paulista, Botucatu, SP, Brasil.

**Keywords:** Anesthesia, Anxiety, Child, Hydrocortisone, Stress, Psychological, Parent-Child Relations, Anestesia, Ansiedade, Criança, Hidrocortisona, Estresse Psicológico, Relações Pais-Filho, Anestesia, Ansiedad, Niño, Hidrocortisona, Estrés Psicológico, Relaciones Padres-Hijo

## Abstract

**Objective:**

The main objective of this study was to compare stress and anxiety levels in children undergoing surgical procedures with or without parental presence at induction of anesthesia by measuring salivary cortisol levels and applying the mYPAS.

**Method:**

Quasi-randomized trial with children aged 5–12 year, with ASA physical status I, II, or III, undergoing elective surgery. According to parents’ willingness, the pair were defined as accompanied or unaccompanied group. Chi-square, Fisher’s exact tests, Student’s *t* test, Mann-Whitney, Hodges-Lehman and Spearman’s tests were used for statistical analyzes.

**Results:**

We included 46 children; 63% were preschool children mostly accompanied by their mothers (80%). The median mYPAS score was 37.5 (quartile range, 23.4–51.6) in unaccompanied children, and 55.0 (quartile range, 27.9–65.0) in accompanied children, with an estimated median difference of +11.8 (95% CI of 0 to 23.4; p = 0.044). There were no significant differences in the mean salivary cortisol levels.

**Conclusion:**

The level of anxiety was higher in accompanied children. There were no differences in salivary cortisol levels between both groups. **Brazilian Registry of Clinical Trials (ReBEC):** RBR-9wj4qvy.

## RESUMO

**Objetivo:**

O principal objetivo deste estudo foi comparar os níveis de estresse e ansiedade em crianças submetidas a procedimentos cirúrgicos com ou sem presença dos pais na indução da anestesia, medindo os níveis de cortisol salivar e aplicando o mYPAS.

**Método:**

Ensaio quaserandomizado com crianças de 5 a 12 anos, com estado físico ASA I, II ou III, submetidas a cirurgia eletiva. De acordo com a disposição dos pais, o par foi definido como grupo acompanhado ou não acompanhado. Foram utilizados testes de qui-quadrado, exato de Fisher, t de Student, Mann-Whitney, Hodges-Lehman e Spearman para as análises estatísticas.

**Resultados:**

Foram incluídas 46 crianças; 63% delas em idade préescolar, principalmente acompanhadas por suas mães (80%). A pontuação mYPAS mediana foi de 37,5 (intervalo interquartil, 23,4–51,6) em crianças não acompanhadas e de 55,0 (intervalo interquartil, 27,9–65,0) em crianças acompanhadas, com uma diferença mediana estimada de +11,8 (IC de 95% de 0 a 23,4; p = 0,044). Não houve diferenças significativas nos níveis médios de cortisol salivar.

**Conclusão:**

O nível de ansiedade foi maior em crianças acompanhadas. Não houve diferenças nos níveis de cortisol salivar entre os dois grupos. **Registro Brasileiro de Ensaios Clínicos (ReBEC):** RBR-9wj4qvy.

## RESUMEN

**Objetivo:**

El objetivo principal de este estudio fue comparar los niveles de estrés y ansiedad en niños sometidos a procedimientos quirúrgicos con o sin presencia de los padres en la inducción de la anestesia mediante la medición de los niveles de cortisol salival y la aplicación del mYPAS.

**Método:**

Ensayo cuasi-aleatorio con niños de 5 a 12 años, con estado físico ASA I, II o III, sometidos a cirugía electiva. Según la disposición de los padres, se definieron como grupo acompañado o no acompañado. Se utilizaron pruebas de chi-cuadrado, exacta de Fisher, t de Student, Mann-Whitney, Hodges-Lehman y Spearman para los análisis estadísticos.

**Resultados:**

Se incluyeron 46 niños; el 63% eran niños en edad preescolar, en su mayoría acompañados por sus madres (80%). La puntuación mYPAS mediana fue de 37,5 (rango intercuartílico, 23,4–51,6) en niños no acompañados y de 55,0 (rango intercuartílico, 27,9–65,0) en niños acompañados, con una diferencia mediana estimada de +11,8 (IC del 95% de 0 a 23,4; p = 0,044). No hubo diferencias significativas en los niveles medios de cortisol salival.

**Conclusión:**

El nivel de ansiedad fue mayor en los niños acompañados. No hubo diferencias en los niveles de cortisol salival entre ambos grupos. **Registro Brasileño de Ensayos Clínicos (ReBEC):** RBR-9wj4qvy.

## INTRODUCTION

Hospitalization is a major stress factor for children and parents, as it implies a break in daily routine and fear of the unknown. The combination of anesthesia and surgery is a common cause of anxiety. It is estimated that 40 to 75% of children have preoperative anxiety related to the feelings expressed by their parents or caregivers^(1–3)^. Because anesthesia induction is the most stressful period, intervention strategies have been investigated to reduce anxiety, such as premedication, psychological preparation, use of recreational activities, use of audiovisual resources, and parental presence in the operating room^(4,5)^.

Stress can be defined as the consequence of physical and mental responses to our inability to distinguish between real risks and personal expectations. It generates anxiety and can affect the nervous, endocrine, and immune systems. Anxiety is an organic response, in the psychological and physiological scope, represented by somatic, emotional, cognitive and behavioral conditions^(6)^.

The modified Yale Preoperative Anxiety Scale (mYPAS) is an observational instrument developed to assess anxiety in children while waiting for surgery in the preoperative waiting area, on entrance to the operating room, and upon introduction of the anesthesia mask. The Hospital Anxiety and Depression Scale (HADS) is a self-assessment instrument that separates the concepts of anxiety and depression in adults, diagnosing mood disorders and somatic illnesses^(7)^.

Exposure to stressful events leads to changes in the central nervous system, autonomic nervous system, and hypothalamic-pituitary-adrenal axis. Over time, there is excessive cortisol secretion, which affects the function of some neurotransmitters, such as epinephrine, norepinephrine, and serotonin, thus altering cognitive function as well as the immune and metabolic systems^(8,9)^. Cortisol levels can be measured non-invasively in saliva and increases with physiologic stress both in adults and children, reflecting higher plasmatic levels of the hormone in this situation^(10,11)^.

The research question is if the parents’ presence at the surgical theater has positive effect over the children’s stress level. Our hypothesis is that the presence of parents during anesthesia induction would decrease the stress levels of children and their parents assessed by behavioral tools and measured salivary cortisol, but the literature is not clear regarding this. Therefore, the main objective of this study was to compare stress and anxiety levels in children undergoing surgical procedures with or without parental presence at induction of anesthesia by measuring salivary cortisol levels and applying the mYPAS. As secondary objective, we also measured salivary cortisol and applied the HADS to parents, present or not during induction, and searched for other potential influencing factors that might contribute to higher hormone levels in them.

## METHOD

### Design of Study

We conducted a prospective, quasi-randomized trial. This paper follows the guideline of the Transparent Reporting of Evaluations with Nonrandomized Designs (TREND) statement.

### Population, Local and Selection Criteria

The study included 46 children undergoing general anesthesia and was performed in the surgical unit of a university hospital which provides services to the Brazilian Unified Health System (SUS), a reference for medium and high complexity care in the state of Sao Paulo.

Were included children aged 5–12 year with American Society of Anesthesiologists (ASA) physical status I, II, or III who were accompanied by a parent and underwent elective surgical procedures from July to December 2019, from 7:00 to 11:30 am. For parents, inclusion criteria were being literate. We did not included children who received preanesthetic medication, who required postoperative mechanical ventilation, and those receiving corticosteroids. We also did not included parents who had a meal within 1 hour of salivary sample collection and those on use of corticosteroids or antidepressants. Since correlations were made, any non-inclusion criteria present in the children and/or the parents meant that the pair would not be enrolled.

### Sample Definition

Only surgeries scheduled for the morning period were included, in respect to the cortisol’s circadian rhythm levels^(12)^. Children and parents have spent the night in the hospital yards. All surgeries were performed at the first surgical schedule.

As our primary objective consisted of two quantitative variables measured in the participating children, so we decided to perform two sample size calculations. On a previous study by Kain et al.^(7)^, the mYPAS anxiety score of preschool children measured rose from a value of 28 ± 8 (no anxiety) in the preoperative holding area to 35 ± 12 upon entering the operating room, reaching 43 ± 15 (denoting anxiety) at the time of facial mask placement. Taking in consideration a α-error tolerance of 0.05, and a detection power of 80% for a difference of 15 points between groups with a standard deviation of 15 in the score value, 17 patients per group would be required for the hypothesis testing. Due to the high circadian variation of the salivary cortisol range among children, we opted to use the standard deviation of three different moments (morning, noon, and night) observed in health children from 1 to 8 years-old by Kiess et al.^(12)^. In this study, a mean value of 3.8 nmol/L for the standard deviation in the evaluated timeline was noted. For a power detection of 80% with a tolerance of 0.05 for type I error, 20 patients would be needed per group to detect a 3.5 difference in the salivary cortisol levels measured in nmol/L. Considering the non-randomized nature of our work and potential losses for attrition, a total sample size of 46 participants were programed to be enrolled in the study.

Two groups were defined according to the parents’ verbalization about their desire to be together with the child during anesthetic induction. Due to ethical reasons, a proper randomization would not be feasible. Since the primary objective of the study concerned the children, a quasi-randomization model was based upon parent’s willingness. Parents provided written informed consent preoperatively while the child waited for surgery in the preoperative waiting room.

### Procedures

Using the Salivettes^®^ kit, we collected saliva samples from parents accompanying their child at the operating room (after induction of the child’s anesthesia), and from those who waited outside the operating room (10 minutes after the child had entered the operating room).

For children we measured their anxiety levels by the modified Yale Preoperative Anxiety scale (m-YPAS) after the group definition. This tool is an observational measure of children’s preoperative anxiety consisting of 27 items divided into 5 categories: activity, vocalizations, emotional expressivity, state of apparent arousal, and use of parents. Its scores range from 23.4 to 100 with higher scores indicating greater anxiety. Cut-off scores to classify children with or without anxiety were: without anxiety (23.4–30), with anxiety (>30)^(1,7)^. All evaluations were performed by a single examiner, who was the same for all children, in the presence or absence of their parents.

We also asked all parents to complete the Hospital Anxiety and Depression Scale (HADS), a self-assessment questionnaire with fourteen items for depression and anxiety (seven for each domain) that has been found to be a reliable instrument in the setting of hospital outpatient clinic. A total subscale score of >8 points out of a possible 21 denotes considerable symptoms of anxiety or depression. We classified parents with a HADS–Anxiety score of 0 to 8 as non-anxious, and those with a score ≥9 as anxious^(8)^. HADS was complete by the parents at the same moment the saliva sample was collected, as described above.

In the operating room, all children received standard hemodynamic monitoring, including respiratory rate, heart rate, noninvasive systemic blood pressure, and oxygen saturation measurements. The accompanying parents remained with their child until the beginning of anesthesia induction.

**Figure 1:**
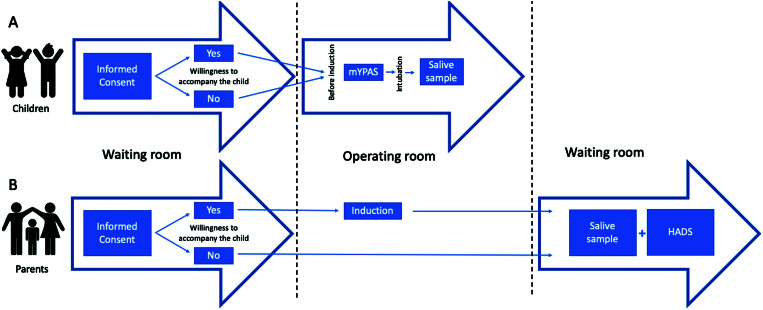
Procedures’ timeline. “A” represents the procedures performed in the included children, while “B” represents the procedures in the parents. Source of vectors: www.br.pinterest.com

After the child had received inhalation induction with sevoflurane via face mask, a single researcher collected saliva samples from all children in both groups by placing a cotton swab on the inner cheek for approximately 20 minutes. We measured salivary cortisol levels using an ELISA kit (GenWay Biotech Inc., San Diego, CA, USA) (Figure 1).

### Data Analysis and Treatment

Prior to statistical analysis, we tested continuous variable distributions for normality using the Shapiro-Wilk test and histogram analysis. For descriptive statistics, qualitative variables are expressed as frequency and percentage, and quantitative variables as mean and standard deviation (SD), when normally distributed, or median and quartiles when non-normally distributed.

We investigated associations between groups and between categorical explanatory variables using a chi-square test or Fisher’s exact test when needed. For between-group comparisons of quantitative variables, we used Student’s *t* test for normally distributed data (presented with the 95% confidence interval for the difference in means). The Mann-Whitney test was performed for non-normally distributed variables with the Hodges-Lehman estimator for treatment difference (estimated median difference between groups and its 95% confidence interval).

For potential influencing factors of higher salivary cortisol levels in the parents, we assessed differences in the whole sample. Spearman’s correlation coefficients were used to assess the relationship of children’s and parent’s continuous and discrete variables with parental salivary cortisol, and Student’s t test for comparisons of this last variable (salivary cortisol level) in dichotomic grouping by sex, and children’s physical status and history of previous surgery. Due to the exploratory nature of secondary objectives, no correction method was used in those last multiple comparisons. Data were analyzed using SAS, version 9.4, and set the level of significance at p < 0.05 for all analyses.

### Ethical Aspects

The study was approved by the review board of the institutional research ethic committee on May 10, 2019 (number 3.318.082) and registered at the Brazilian Clinical Trials Registry platform (ReBEC, number RBR-9wj4qvy). The research was performed is in accordance with the Brazilian government determination number 466, from 2012. Informed consent, including the potential benefits of the study results, was obtained of all parents of included children. The child’s assent was obtained when the researcher perceived cognitive and emotional maturity for such. The child was approached, and after a brief explanation of the procedures in a simplified manner, asked if they agreed to participate, following prior authorization from the parents.

## RESULTS

**Figure 2:**
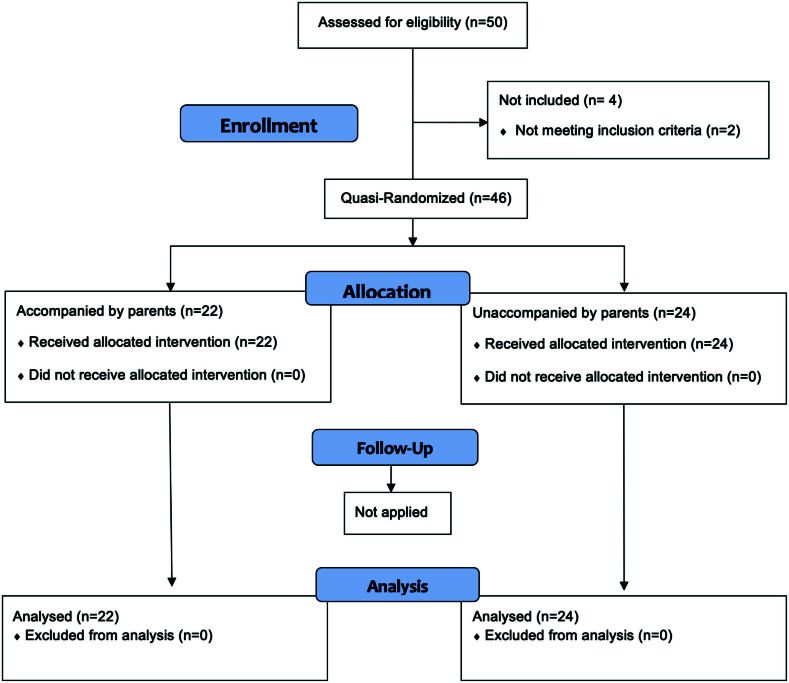
Flowchart of participants’ recruitment.

The study included 46 children undergoing general anesthesia, divided into 2 groups: accompanied group (n = 22) and unaccompanied group (n = 24) (Figure 2). Table 1 shows child and parent sociodemographic data. Preschool boys with an ASA physical status of I accounted for most of the sample. As for the accompanying parent, the mean age was 33 to 34 years, mostly mothers with high school diploma.

**Table 1:** Distribution of demographic characteristics of groups of children accompanied and unaccompanied by parents. The results show children and parents data – Botucatu, SP, Brazil, 2019.

Variable	Accompanied group (n = 22)	Unaccompanied group (n = 24)
Child age (months)^†^	44 ± 26	36 ± 29
Sex^‡^		
Male	14 (63%)	13 (65%)
Female	8 (36%)	7 (35%)
Weight (kg)^†^	17 ± 9	12 ± 6
ASA^‡^		
1	13 (59%)	9 (45%)
2	7 (31%)	7 (35%)
3	2 (9%)	4 (20%)
Schooling^‡^		
Preschool	12 (54%)	15 (75%)
School-age	10 (44%)	5 (25%)
Previous surgery^†^		
Parent age (years)*	34 (31/37)	33 (29/38)
Parent^‡^		
Father	5 (22.7%)	4 (16.6%)
Mother	17 (77.3%)	20 (83.3%)
Level of parent’s schooling^‡^		
Completed Elementary school	9 (40.9%)	8 (33.3%)
Completed High school	11 (50%)	10 (41.6%)
College education	2 (9.1%)	5 (20.8%)

^‡^Values expressed as absolute and relative frequency;

^†^Values expressed as mean and standard deviation.

*Values expressed as median and quartiles.

Table 2 shows the differences in salivary cortisol levels and anxiety scores in children (mYPAS) and parents (HADS) according to group. The mYPAS scores differed significantly between accompanied and unaccompanied children (p = 0.044), with higher anxiety scores (median, 55.0; IQR, 37.9–65.0) for children with parental presence at induction of anesthesia. Although children accompanied by a parent also had higher salivary cortisol levels, there was no statistically significant difference between groups.

**Table 2:** Differences into mean and median in salivary cortisol levels (nmol/L) and anxiety scores in children (mYPAS) and parents (HADS) according to group – Botucatu, SP, Brazil, 2019.

Variable	Unaccompanied group n = 24	Accompanied group n = 22	Difference between means and medians (95% CI)	p
Salivary cortisol in children^†^	5.18 ± 3.13	7.40 ± 5.97	–2.22 (–6.19 to 2.29)	0.258
Salivary cortisol in parents^†^	2.29 ± 1.56	2.80 ± 1.53	–0.50 (–1.61 to 0.59)	0.354
mYPAS*	37.5 (23.4/51.6)	55.0 (27.9/65.0)	–11.8 (–23.4 to 0)	0.044
HADS – Anxiety*	11.0 (6.0/13.5)	9.0 (5.7/12.0)	1.0 (–1.0 to 4.0)	0.256
HADS – Depression*	7.0 (4.0/10.0)	7.0 (3.0/9.2)	0 (–2.0 to 3.0)	0.800

^†^Values expressed in nmol/L as mean and standard deviation – Student’s *t* test;

*Values expressed as median and interquartile range – Mann-Whitney; HADS – Hospital Anxiety and Depression Scale.

Table 3 shows the correlations of child and parent variables with parental salivary cortisol levels. A statistically significant correlation was found with mYPAS scores (r = 0.396; p = 0.022) and HADS–Depression scores (r = 0.345; p = 0.049).

**Table 3:** Correlations of children and parents’ variables with parental salivary cortisol levels (Unaccompanied group n = 24; Accompanied group n = 22) – Botucatu, SP, Brazil, 2019.

Variable	Correlation coefficient	p
Child age*	0.013	0.941
mYPAS	**0.396**	**0.022**
Parent age	0.073	0.688
Parent level of education*	0.014	0.938
Parent subjective feeling*	–0.013	0.941
Hospital Anxiety and Depression Scale – Anxiety	0.198	0.269
Hospital Anxiety and Depression Scale – Depression	**0.345**	**0.049**

*Spearman’s correlation.

mYPAS – modified Yale Preoperative Anxiety Scale.

Parent level of education: 1 – Elementary/middle school (with or without diploma); 2 – High school (with or without diploma); 3 – College (with or without degree).

Parent subjective feeling: 1 – Very bad; 2 – Bad; 3 – Regular; 4 – Well; 5 – Great.

We also analyzed the parental salivary cortisol levels according to parent sex (2.14 ± 1.66 for males; 2.61 ± 1.54 for females; p = 0.514), child ASA physical status (2.65 ± 1.72 for ASA 1; 2.39 ± 1.37, for ASA >1; p = 0.639) and child’s history of previous surgery (2.72 ± 1.45 for no previous surgery; 2.21 ± 1.68, for those with previous surgery; p = 0.363) did not show any statistical significant results for these variables.

## DISCUSSION

This study had compared the levels of anxiety and stress in children with or without the presence of parents at induction of anesthesia, measuring the levels of salivary cortisol and applying mYPAS and HADS. The level of anxiety determined by the mYPAS was higher in accompanied children and correlated positively with the parent’s salivary cortisol level. There were no differences in salivary cortisol levels between both groups of children and parents.

We chose mYPAS to assess anxiety in children as it is an easy-to-apply observational instrument, avoiding the stress of interaction between researchers and children. This scale has a sensitivity of 85% and a specificity of 92% for assessing anxiety in preschool and school-age children. We used the HADS to evaluate parental anxiety after the child entered the operating room because it is a quick self-assessment scale that is easy to fill out without the help of a researcher. This scale includes psychic and physical symptoms, in addition to separately measuring anxiety, an emotional disorder often observed in daily practice^(8)^.

Our results showed that, with or without parental presence during induction of anesthesia, all children were anxious; however, higher anxiety scores were observed in the accompanied group. All parents were also anxious, whether present or not during induction. This can be explained by several factors within the context of family anxiety that serve as predictors of preoperative anxiety in children. These factors include fear of the unknown, separation from parents, preschool age, lack of information provided by the health care team about the procedure to be performed, the non-use of non-pharmacological methods, and the presence of anxious parents. All of which can directly impact child behavior and cooperation during induction^(2)^.

Although other authors have found a correlation between the levels of parents’ anxiety and children’s stress during induction, we could not find the same results^(13)^. Also, differently from ours findings, a study reported that children with parental presence during induction were less anxious than unaccompanied children^(14)^. Intriguingly, the premedication may be more effectiveness on reducing the anxiety of the children and of the parents than the parents’ presence during induction^(2,15,16)^. Psycological preparation of children has also been proven to be effective on reducing preoperative anxiety^(6)^.

The child age factor can increase preoperative anxiety. One interesting research compared the level of preoperative anxiety in preschool vs school-age children and concluded that preschool-age children had higher levels of anxiety than older children due to limited understanding of the purpose of anesthesia/surgery, thus feeling threatened by the procedures performed. School-age children, however, were more compliant because they are emotionally independent from their parents and able to understand the benefits of anesthesia and surgery^(17)^. Other factors, beside de child age that may influence the stress levels in the patient are: previous surgery/anesthesia, ambulatory surgery, parent’s stress level; and, for the parents, the cardiac surgery is a main stress source^(18)^. In our study we have not studied the specific age groups impact on the stress of included children.

There is a small number of included children classified as ASA III physical status in our sample, what does not allow us to conclude on the influence of the physical status on the anxiety level of them. Other factors, as pre-scholar age and previous medical encounters, may predict the presence of anxiety, but the ASA physical status has not been reported as one factor^(19)^. We did not study the influence of ASA physical status on parents’ stress level either.

One study analyzed children’s behavior and emotional state and parents’ anxiety at induction of anesthesia. They found association between preschool-age children’s anxiety levels and anxious parents, with a considerable impact on the correlation of both child and parent behaviors. In this study, more anxious parents make calm children also anxious, which can affect the child’s behavior and postoperative recovery, thus negatively influencing future procedures. In addition, parents’ anxiety can be harmful to the child’s development^(20)^. Also, age of child, parent’s gender, information about the anesthesia, fear of postoperative pain, and parent’s occupation are considered parent’s stress factors^(21)^. These results cannot be compared to ours, since all parents in our sample presented a high level of stress. This may have negatively influenced the accompanied children, who presented higher stress level in our study.

Other researchers also investigated whether parental presence during induction of anesthesia would reduce the child’s preoperative anxiety and concluded, like our study, that the presence of an anxious parent can increase anxiety in a calm child and further increase the stress of anesthesia induction in an anxious child. Therefore, parents should have access to information and guidance on the anesthetic/surgical procedure to help their children remain calm during induction^(22)^.

In the present study, the high prevalence of anxiety in parents may be attributed to the fact that the sample consisted mostly of women, who tend to be more anxious than men, or at least express it more often, due to cultural differences^(7,21)^. Some psychological theories suggest that mothers play an important role in their child’s hospitalization process, being more emotionally involved than fathers, in addition to the mother-child bond formed during pregnancy^(21,23)^.

A Cochrane review that included 28 trials with 17 interventions, of which five investigated the effect of parental presence at induction of anesthesia, refuted the results of many studies by stating that parental presence during induction has no benefit in reducing children’s anxiety, like our finds. The review also concluded that the preoperative use of pharmacological methods, such as anxiolytic medications (oral midazolam), is more effective at reducing children’s anxiety^(24)^.

We also assessed parents’ and children’s level of stress by measuring salivary cortisol, as it is a rapid, easy, noninvasive method. Cortisol is a glucocorticoid secreted by the hypothalamic axis into the bloodstream that becomes readily detectable in saliva under physical or psychological stress. In our study, cortisol levels were higher in children than in parents.

Even though statistically significant positive correlations between parental salivary cortisol and children’s mYPAS score as well as parent’s HADS score for depression were observed, the coefficient for these correlations were low. One possible explanation for this occurrence is the high variability in cortisol levels between and within individuals due to multiple reasons that includes not only physical and/or psychological stress, but also circadian oscillations of the hormone, which invariable affects its salivary concentration^(12)^. This finding is consistent with other published works on the matter and may explains why the determination of a clear cutoff value of cortisol levels as a surrogate biomarker for stress is notoriously elusive in the literature. A recent study on child undergoing congenital cardiac surgery have found that salivary cortisol levels can be considered a predictive indicator of anxiety on pre- and postoperative period^(25)^.

Children tend to be more anxious than adults and that increased cortisol levels are associated with the child’s fear of discomfort, as well as lack of information, about the procedure to be performed, thus leading to greater anxiety^(25)^. For these reasons, in children, is important the psychological preparation with verbal explanations, informative leaflets and videos, and visual illustrations to reduce anxiety^(26)^.

Research that analyzed factors that influence the salivary cortisol concentration and response, emphasized that environmental and behavioral factors interfere with salivary cortisol measurement. It is also stated that sleep deprivation and preoperative fasting act as a stress mechanism, which increases cortisol production; therefore, younger children (who need more meals and more hours of sleep daily) have elevated cortisol levels^(10)^. The age of our children sample did not differ among the groups and did not influence the salivary cortisol levels analysis. Also, the age range of included children in our sample is categorized at the same cluster to analysis the daily cortisol variation in his study^(12)^.

In general, parents tend to fast together with their child, further increasing their stress mechanism. Some authors have reported that children undergoing frequent surgical procedures are exposed to high levels of stress and anxiety and described the relationship of cortisol secretion with the child’s immunity, as the immune system is related to neuroendocrine processes that affect antibody secretion, T-cell function, and macrophage and eosinophil reactivity^(11)^.

Parents’ anxiety influences children’s anxiety, especially in young mothers with children under 5 years of age, who report greater anxiety because their children cannot communicate clearly. Parents with a lower level of education have an increased level of anxiety as they lack knowledge and have difficulty understanding the instructions received, thus negatively affecting anxiety^(3)^. The anxiety level of parents tends to be similar to that of children, and the higher the parents’ level of education, the greater their level of anxiety^(27)^. Conversely, there are reports that the higher the parents’ level of education, the lower their anxiety scores^(22)^. Our results, however, did not showed such correlation. According to other study, maternal anxiety is related to the child’s anxiety status, with no relationship between maternal education level and anxiety level^(28)^. Our results showed a correlation of both mYPAS and HADS scores with parental salivary cortisol levels, where the children of more anxious and distressed parents were also more anxious. Elevated cortisol levels trigger harmful psychosomatic reactions^(29)^.

The incorporation of educational interventions has had a positive impact in Brazil and in the world, recognizing the importance of identifying and accepting the needs arising from patients and families^(15,30)^.

In the present study, parents reported that they felt very distressed even being present during their child’s induction. Other study conducted by our group have concluded that, when accompanying their children during hospitalization and a subsequent surgical procedure, parents experience feelings of uncertainty, in addition to the transmission of anxiety from parent to child. Parental anxiety can be identified at the time of separation from children, by seeing them being anesthetized, as well as in relation to their concern about the child’ pain and recovery^(2)^.

This study has some limitations. It was conducted in a single hospital that serves a population of adults and children; therefore, there was no specific preoperative room for the children and their parents. The group allocation may have influenced the outcome, since parents of anxious children maybe incline to accompany them to the operating room. Also, we did not follow up children or parents in the postoperative period, did not have studied the influence of children’s ASA physical status and age on the parents and children’s stress level. In addition, although salivary cortisol is a biomarker that can be measured quickly, easily, and noninvasively, its levels vary widely in the literature, thus making it difficult to compare the results with normal cortisol levels. Findings referring to the secondary objective of our study must be viewed with caution due to the inherent exploratory nature of them (absence of power detection assessment and correction method for multiple comparisons).

## CONCLUSION

There was no difference in salivary cortisol levels between accompanied and unaccompanied children. However, the level of anxiety determined by the mYPAS scale was higher in children with parental presence at induction of anesthesia. Parent’s anxiety was high in both groups, regardless of their presence at induction, and their salivary cortisol levels correlated positively with higher values of children’s anxiety measured by the mYPAS score and with signs of depression in the HADS score.
